# Making mapping matter: a case study for short project international partnerships by global public health students

**DOI:** 10.3402/gha.v7.23593

**Published:** 2014-06-24

**Authors:** Rosemary Wyber, James R. Potter, Jennifer B. Weaver

**Affiliations:** Department of Global Health and Population, Harvard School of Public Health, Boston, MA, USA

**Keywords:** sanitation, GIS, mapping, education, global

## Abstract

**Background:**

A large number of global public health students seek international experience as part of their academic curriculum. These placements are often short, given the constraints of cost and time available within the academic calendar. In contrast to international electives for clinical students there are few published guidelines on practical, ethical or feasible projects. This paper describes a ten-day sanitation mapping project in Mumbai, India and explores the broader implications for global public health student electives.

**Methods:**

Three graduate public health students conducted a geographic review of sanitation facilities in Cheeta Camp informal settlement, Mumbai. Forty-six toilet blocks with 701 individual seats were identified. The project was reviewed ethically, educationally and logistically as a possible model for other short-term international projects.

**Conclusions:**

Clearer guidelines are needed to support non-clinical placements by global public health students. Projects that are feasible, relevant and meaningful should be foster maximise benefit for learners and host communities.

Educational engagement in global health issues has crystalized into a unified academic discipline over recent decades (1). Increasingly, clinical training for medical, nursing and midwifery students includes or encourages some period of international clinical experience (2). Approximately 40% of medical students from North America and the United Kingdom travel overseas for international medical electives (3). These projects have shortened in duration as international air travel has become more affordable and curricula have become crowded with subspecialties (4). The growing number of international placements has spurred an increasingly strong body of literature exploring practical and ethical issues of international clinical experience. Best practice guidelines have been proposed with a focus on strong program structure, clear expectations, and pre-departure training (5).

However, interest in global health extends beyond clinical programs. Education in global public health continues to expand in scope and scale. To date, 32 of the Council on Education for Public Health (CEPH) accredited schools in the United States offer a Global Health concentration (6). The majority of these programs include an international placement, practicum, or course. Public health is an area of significant worldwide workforce shortage, necessitating a considered approach to training, development, and knowledge exchange (7, 8). Yet, global public health placements are poorly examined in contrast to clinical electives in low- and middle-income countries. Numbers of students, length of visits, destinations, relationships with host communities, and outcomes are largely unknown – and best practices have not been clearly defined.

Public health education differs in several important ways from training in a clinical discipline. Students of public health enter into short-term projects with a disparate array of previous experience and skills, and while some may have undertaken public health courses, they often have not had the standardization of skills, socialization, and clinical experience to which medical students are exposed prior to departure. They are also less supported by literature on the ethics, best practices, and conventions of global health than their clinical colleagues (9). Short-term projects often focus on knowledge development though observational projects or data collection. Although these may not be formal research activities, potential risk and benefits are comparable. Student audits, pilot or investigative projects warrant ethical consideration even when there is no intent to publish or disseminate results (10). Global public health placements are generally community based, reflecting health needs at a population level. The immersive nature of these community projects is substantively different to the clinical electives in global health. Clinical placements are generally based in established institutions with a focus on service delivery for individuals. However, population experiences may occur in a disparate array of settings, with a broad range of local collaborators and potentially significant implications for large numbers of people. Despite this variability, best practice guidelines are far better developed for clinical than population level global health placements. Clearer expectations about the role and remit of international student experiences in population health are required. Statements by professional bodies should address the delicate balance between creating learning opportunities for students and the address the actual and stated needs of the local populations. Clear examples of appropriate projects that address the priorities of host communities and provide safe, relevant, and ethical student experience in public health should be identified.

This paper describes a 10-day sanitation mapping project conducted in an informal settlement in Mumbai, India. This proof of concept project is used to illustrate ethical, educational, and practical considerations for global public health placements and highlight the potential role of mapping projects. Broader considerations about international placements in global public health training are also explored.

## Methods

Twelve students from the Harvard School of Public Health participated in an Urban Health internship hosted by the Tata Institute of Social Sciences (TISS) in Mumbai, India (11). The project lasted for 18 days in January 2012. Six of the students were based in Cheeta Camp informal settlement, a 36-year-old settlement with a population of approximately 117,000 people (11). The first 3 days of the trip were devoted to introductory lectures, a tour of Cheeta Camp, and meetings with non-government organizations (NGOs) and government health providers. Students then broke into groups of three to identify areas of interest and develop an area of focus and a short exploratory project. After discussions with translators and community members, the group that will be the focus of this paper focused on sanitation. Following a brief literature review, the group developed a project to identify all public toilet sites in Cheeta Camp. Students planned to locate toilets, plot GPS co-ordinates, and collect data about the cost and condition of each toilet block. Sanitation mapping was selected to represent apparent interests of the community and as a discrete project able to be completed in a short time frame. The project was discussed with academic staff and community representatives at the host institution TISS and considered suitable to proceed without formal ethics review.

Mapping of toilet blocks began on the fourth day, based on an informal hand drawn map provided by the local government health post. Using this map as a guide, students were assisted by a local community translator to find public toilets in each sector of the camp. Over the following days, the students sought out toilet facilities and mapped their location using a GPS smartphone application. Details of each toilet were recorded: open/closed, number of seats, provision of water and lighting, cost and opening hours. Qualitative data on the history of the toilet and approximate building and renovation dates were also recorded. The students entered all data into a spreadsheet at the end of each day and plotted coordinate points in Google Maps. The seventh and final field day was spent revisiting some areas and validating the Google Map with physical locations on the ground. At the conclusion of the project, the students presented their findings to colleagues at TISS. Color posters of the maps were printed and students were able to revisit initial informers and provide copies of the poster. Copies of the map and findings were also provided to local city officials. The maps were generally well received, although no formal follow-up of opinions or outcomes has been possible to date.

## Results

Overall, 46 toilets blocks with 701 ‘seats’ were identified in Cheeta Camp. Eight (17%) of these toilets were closed during the period of the survey. Of the 38 operational toilet facilities, 15 (40%) were free and generally did not supply water or lighting at night. A further 23 toilets (60%) charged nominal fees, which generally amounted to 1–2 INR per use, or a monthly pass system for some families living close to the facility. These ‘pay’ toilets generally provided lighting and water, though many close for several hours during the night. Toilets were distributed throughout the camp, though illegal ‘encroachment’ areas had noticeably few toilet facilities. An interactive map of the toilet blocks, including photos and reported details is available online at https://maps.google.com/maps?hl=en&tab=ml

The process of identifying toilet locations and establishing conditions in each facility provoked considerable discussion with community members and bystanders. These discussions were conducted between one Hindi-speaking student and paid, untrained translators assisting with the urban health internship. Several recurring themes emerged. First, there was a strong preference for pay toilets because they were perceived to be safer, cleaner, and better maintained. Second, bystanders indicated that service provision to toilet facilities was sometimes complicated by elections; the opening of a toilet block or provision of water and lights was often used as an election promise but frequently not delivered after elections. Finally, there was a strong informal consensus that not enough toilets blocks were provided to meet the needs of the community. The students were often asked whether this survey would be instrumental in the development of more toilet facilities – a question difficult to answer without locally accessible partners to whom students could consult.

During the data collection, students were able to make other informal sanitation observations. In particular, it became clear that a wide range of stakeholders had been responsible for building and maintaining toilet blocks. The Maharashtra state government, the Municipal Corporation of Greater Mumbai, individual politicians, and multiple non-profit donors had reportedly provided toilets; yet, it was unclear how these organizations communicated for sanitation planning. In addition to mapping toilet blocks, students observed considerable open defecation, particularly by children. Bystanders reported open defecation as the norm for children until the age of five or six. Residents expressed that the practice was convenient (families did not need to leave household tasks or other children for toileting), did not involve any fees, and did not expose children to the perceived risk of falling into toilet pits.

In addition to sanitation mapping, time in Cheeta Camp enabled students to observe a range of other daily activities and habits with health relevance, including water collection, cleaning, food preparation, litter, and livestock management. While these areas were not formally recorded or investigated, the experience provided a basic introduction to daily life in informal, high-density urban living.

## Discussion

Sanitation mapping of an informal urban settlement can be an exercise rich in skills, experience, and outcomes for students, institutions, and host communities. These considerations and outcomes fall into three key domains: benefit to the host community, educational outcomes, and ethical considerations.

### Benefits to the host community: mapping can matter

Infrastructure maps of resource-poor settings have become more common in recent years as technical requirements have become more accessible. This increased technological capacity has been successfully harnessed by academics and policy makers, and to a lesser extent for grassroots democracy advocacy (12–14). A number of NGOs have mapped toilet locations and successfully parlayed this knowledge into a strong advocacy mandate. In a recent example, Transparent Chennai mapped 49 toilets on behalf of IFMR Research and identified similar issues to this project: under-used toilets, no clear lines of accountability for maintenance of public toilets, and limited budgetary allocations (15, 16).

Map-making, in the first iterations of GIS, was dominated by highly technical projects driven by the global north (14). More recently, however, an evolution of functionality and ownership has occurred. Google Maps as a deconstructed form of GIS is free, easy to use, and readily shared online, complemented by practical advice for application (14, 17). Given the availability and user-friendly nature of new mapping technologies, students may be able to work with communities to identify suitable mapping targets and exchange technical for local knowledge. For the purpose of knowledge sharing, free, open source, online software with little hardware requirement is one way of minimizing technological disparity (18).

Aligning with local priorities and research agendas is a challenge for international investigative projects (10). In this example, student conversation with host academics and with community members identified sanitation as a priority area in Cheeta Camp. Despite this informal consensus about the priority problem, there were few suggestions about how students could contribute to tackling the issue. In Cheeta Camp project, no individual or institution made a specific request for geographic data with the project largely devised by students. However, local doctors and medical staff suggested that mapping would be interesting or helpful. This positive feedback may well have been considered a polite or appropriate response to visitors.

The real value of the project to Cheeta Camp is unclear. Printed copies of the map were produced on the last day of the project and distributed by students to the local health center, non-profit groups, and key informants. These copies were received enthusiastically. An online version of the map has received more than 37,000 views. A more robust plan to disseminate and use findings may have been possible if mapping had been offered as a suite of student ‘services’ to stakeholders prior to arrival.

An advantage of mapping projects is that they can be tailored to an array of issues by documenting different items, namely water sources, health care providers, drug dispensaries, or education facilities. This flexibility allows students to have pre-departure mapping training and then adapt the project to address the needs of the host community on arrival. Targets for mapping projects should be identified by local partners to reflect research, policy, or advocacy priorities. Services or infrastructure, which may be modifiable through legislation or altered resource allocation, may be most appropriate. For example, making the map of toilet facilities in Cheeta Camp accessible to international not-for-profit groups contemplating new sanitation construction could improve equity of toilet access. Conversely, some subjects may be unsuitable for student mapping given concerns about safety or unintented consequences. Inappropriate mapping subjects conceivably include illicit drug sales, location of sex workers, or information on individuals or small family groups.

### Educational outcomes

Map-making provides students with a relatively safe immersion experience and fosters development of practical, academic, and interpersonal skills. The initial phase of an idealized mapping project would identify any relevant baseline data. This may require contacting local community groups or government service providers to obtain pre-existing maps and approximate expected number of resources in question. Contacting and negotiating assistance from local stakeholders is a common first step in international health programs, and mapping projects allow students an opportunity to gain experience in this area. This engagement with local stakeholders should be complemented by a literature review to identify comparable international examples. Limited time, resourcing and, infrastructure to complete this review may particularly be challenging in short international projects. For example, in this mapping project, there was little opportunity to explore the kinds of parameters relevant to sanitation audits, including lighting, metrics of cleanliness, or technical toilet pit specifications. After gathering information, students must spend time in the field to find and document points of interest. This field time is well used; students have a clear goal and enough flexibility to spend time simply being in the community. Mapping forces students to venture away from health centers into less familiar parts of a community. Technical GIS, mapping, and data validation skills can also be fostered among students and ideally with local partners.

The mechanics of travelling to an international location, meeting local partners, adapting to new cultural norms, working with translators, and feeding back the results of a mapping project are all critical career skills in global public health that often cannot be readily simulated at home institutions. Short-term mapping projects provide a vivid ‘ground level’ perspective of the social determinants of health (19). Project-directed exploration of these social and economic contributors to health outcomes may also add value to the experiences of medical and allied health students. Moving beyond the comparatively familiar clinical setting provides an opportunity to better understand the social and environmental contributors to ill health.

### Ethical considerations

Short-term global health education immersion is fraught with challenges, and educational mapping projects are far from immune from methodological weakness and unintended consequences. Students may lack the experience, language, and technical skills to partner successfully with local government or NGO staff. Ongoing supervision and mentoring of international students falls outside the remit of these organizations and can strain human resources.

Equally, communities should not be expected to absorb and accommodate the educational desires of visiting students without tangible benefits. In the project described above, students identified significant ‘survey fatigue’ within the community, a phenomenon that has been well documented in social sciences literature (20), but takes on important ethical considerations in the context of short-term educational projects. Residents expressed frustration that surveys, although frequently conducted in their communities, often resulted in no tangible improvement in local conditions. Engaging more substantively with local partners predeparture would provide greater scope for follow-up, map-based research, and advocacy to ensure that mapping projects address the needs and desires of the community. This engagement may also help identify areas for cross-cultural exchange.

Finally, the potential for negative impacts on local communities is omnipresent in all research and observational activities. In addition to survey fatigue, students must be cognizant of local political arrangements and any negative social or political consequences that could affect community members who speak frankly. This is particularly important if items being mapped bear any political significance, for example, if toilets are used as political bargaining chips. Students should discuss potential harms well in advance of the project and be assisted in devising strategies to minimize risk. Similarly, predeparture training to maximize the accuracy of GPS/GIS data collected would make similar projects more valuable. Finally, reflection on the experience and lessons from cultural immersion should occur during the project and act as an adjunct to formal studies at student's home institutions.

These ethical issues are by no means limited to mapping projects. Rather, they appear frequently throughout the field of global public health. As a result, careful consideration of these issues will not only benefit the immediate project but also instill in the students the importance of these considerations throughout their career.

## Conclusions

Global public health placements are underdocumented and analyzed compared with international clinical electives. Detailed consideration of the strengths, weaknesses, and needs of public health students travelling overseas is required from educators, participants, and host communities. Reflective, illustrative examples of short-term public health projects are needed to inform discussion. This overview of mapping sanitation facilities demonstrates some of the unique issues in global public health education.
